# Recommendation of fiducial marker implantation for better target tracking using MV imager in prostate radiotherapy

**DOI:** 10.1002/acm2.12390

**Published:** 2018-06-26

**Authors:** Tianjun Ma, Joshua Kilian‐Meneghin, Lalith K. Kumaraswamy

**Affiliations:** ^1^ Department of Radiation Medicine Roswell Park Comprehensive Cancer Center Buffalo NY USA; ^2^ Medical Physics University at Buffalo Buffalo NY USA

**Keywords:** EPID, fiducial marker, MV imager, prostate, tracking

## Abstract

**Purpose:**

The aim of this study was to develop a model that optimizes the fiducial marker locations in the prostate to increase detectability of the markers in the projected EPID images during VMAT treatments.

**Methods and Materials:**

The fiducial marker tracking capability for each arc was evaluated through a proposed formula. The output of the formula, a detectability score, was calculated with the in‐house developed software written in MATLAB (The Mathworks, Inc., Natick, MA, USA). Three unique weighting factors were added to penalize the detectability score. The detectability scores of four different patterns containing 40 combinations of simulated fiducial marker locations were evaluated with 101 previously treated prostate treatment plans (containing 202 individual arcs). The results were analyzed for each pattern group and each marker separation distance on the transverse plane.

**Results:**

The maximum detectability of the markers occurred when they were placed between 10 and 15 mm from the center of the prostate in the transverse plane and 6–13 mm in the superior–inferior direction. The detectability decreased when the markers were placed beyond 20 mm in both directions.

**Conclusions:**

The fiducial marker‐based detectability score can be used to predict the real‐time tracking capability. Suggestions for optimal insertion locations were given to improve prostate motion management using MV imaging.

## 1 INTRODUCTION

The motion of prostate has historically been an issue in radiation therapy.^1^ Since the implementation of Intensity Modulated Radiation Therapy (IMRT) technique, dose has fallen more rapidly at the boundaries of targets, which makes it increasingly crucial to minimize and monitor the prostate motion during the treatment delivery, especially for cases that are to be treated with SBRT techniques.

Real‐time prostate motion management techniques have been studied for almost a decade.^2^, ^3^, ^4^ Initially, the fiducial markers implanted inside the prostate served as a surrogate for precise alignment of prostate using portal imaging. In most of the recently developed techniques, fiducial markers were used as a prostate margin tracker either by MV, kV or kV‐MV combined images.^2^, ^3^, ^4^, ^5^, ^6^, ^7^, ^8^, ^9^, ^10^, ^11^ It is important to note that the use of kV imaging techniques is not available in all clinics, and the additional dose that would be applied for every treatment fraction is of concern as well. Specialized equipment such as Calypso system (Calypso Medical Technologies, Inc., Seattle, WA, USA) was also used, but not as widely as radiopaque fiducial markers.

With the help of the MV imager, real‐time target localization can be achieved by tracking the implanted fiducial markers without added dose to the patient. However, there are two major fundamental challenges with MV‐based fiducial marker tracking techniques for VMAT deliveries: (a) frequently blocked radiation fields by the multileaf collimator (MLC) and (b) potential large time intervals between two detections.^8^ There should be some optimum way to maximize the possibility of being detected as a real‐time target tracker, as well as serve its original purpose of being a prostate surrogate.

Thus, in this study, we aim to develop a model based on several simulated fiducial marker locations in the prostate to identify the optimal marker locations for MV EPID‐based tracking during VMAT treatments. In addition, we propose a detectability score to predict the detectability for MV‐based fiducial marker tracking techniques and recommend locations within the prostate to implant the fiducial markers to increase detectability of the prostate during treatments.

## 2 MATERIALS AND METHODS

The first part of the study focused on developing a model based on several simulated fiducial marker locations. The origin of imaginary fiducial marker locations was positioned in the center of patient prostate, which was at the center of the MLC field. DICOM RT plan files from previously treated patients were used to generate the detectability score as described in the following sections.

### 2.A Fiducial marker locations

**Figure 1:**
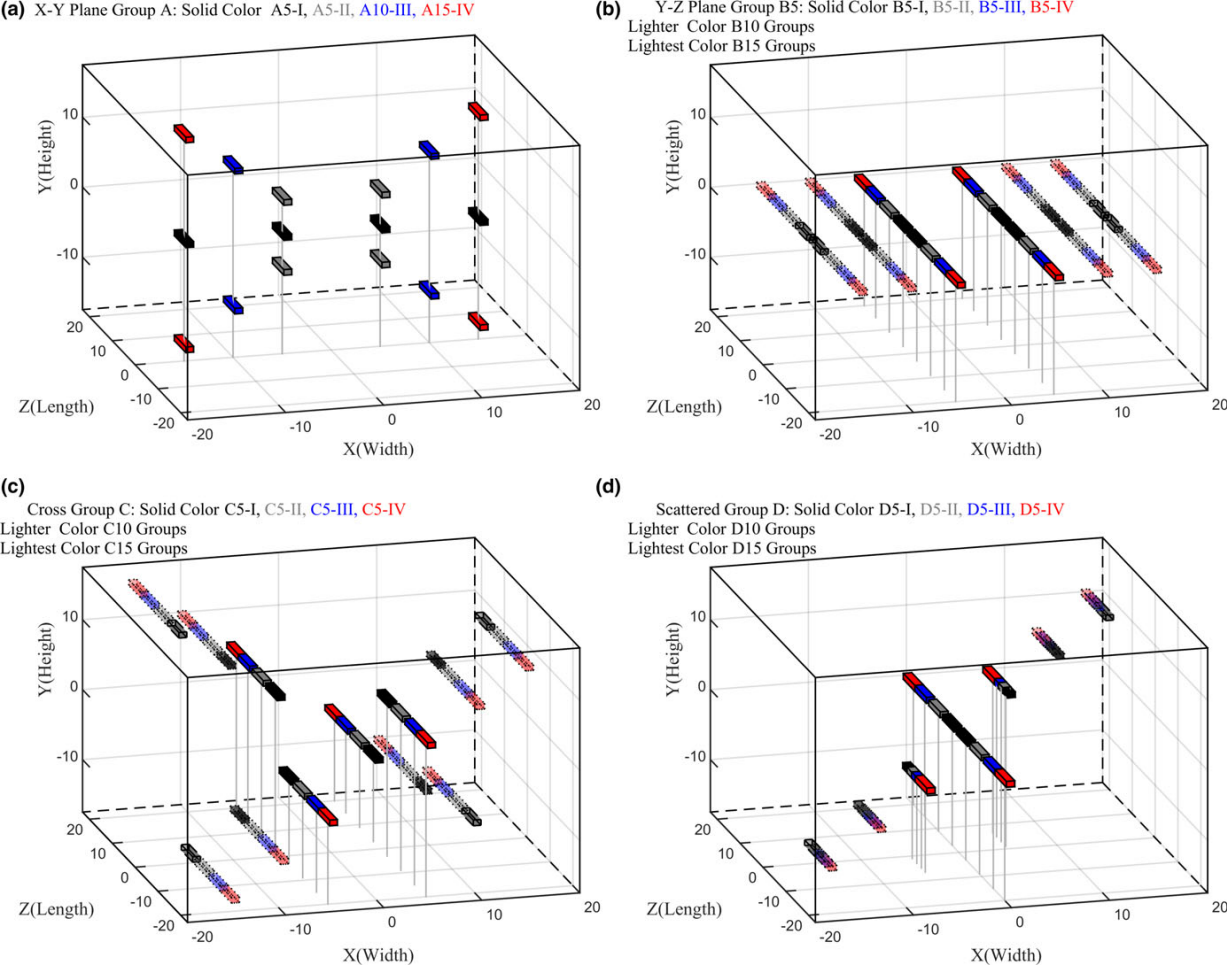
Fiducial marker location demonstration. Each subplot shown the examples of the corresponding pattern group, in which subgroup I was plotted in black color, subgroup II: gray, subgroup III: blue and subgroup IV: red. Different X‐Y plane separations (“a” value) were demonstrated in various transparencies. In subplot D, partial length indicated that the markers were partially overlapped with the adjacent ones.

The radiopaque fiducial marker used in our institution, having a size of 0.8 × 0.8 × 5 mm^3^, was modeled as a 2 × 2 × 6 mm^3^ cuboid volume due to the scatter on the CT images. Typically, in our institute, four fiducial markers are inserted into the patient via an ultrasound‐guided needle and a grid template (CIVCO Medical Solutions, Coralville, IA, USA), a tool for pinpointing the prostate location in transverse plane to accurately deposit the marker into the designated spot. Throughout this manuscript, X‐Y defines the plane parallel to the template plane (transverse plane), from left to right (X) and posterior to anterior (Y) directions, accordingly. The inferior–superior direction is indicated by letter Z. In the X‐Y plane, the resolutions of the fiducial marker center locations were generated according to the resolution of grid template, which is 5 mm, while interval along the Z direction was determined by the size of the fiducial markers and the average‐sized prostate.^12^, ^13^ Total of 40 different fiducial marker implant patterns for four fiducial marker combinations were studied. The patterns include 16 simple planar cases and 24 combinations of scattered cases categorized into four groups (A, B, C, and D). Each pattern group is divided into four subgroups (subgroup I through subgroup IV), as shown in Table 1. To elaborate on the assignment of each group, the location identification for each fiducial marker, and their locations were given as a pattern group letter, followed by parameter “a” value (X‐Y plane separation) and a subgroup number after the dashed line at the end. Example of fiducial marker locations in three‐dimensional space is shown in Fig. 1.

**Table 1:** List of all the locations for the 40 fiducial marker patterns and their corresponding groups/IDs (unit in mm for parameter “a” and “z”)

Format	Pattern (X,Y,Z)	Minimum separation on X‐Y plane (a)	Marker location identifier			
			Group I	Group II	Group III	Group IV
			*z = 0*			
X‐Y Plane Group A	[−3a,0,0; −a,0,0; a,0,0; 3a,0,0] [a,a,0; −a,a,0; a,−a,0; −a,−a,0]	5 (A5)	A5‐I	A5‐II		
		10 (A10)			A10‐III	
		15 (A15)				A15‐IV
			*z = 3*	*z = 9*	*z = 15*	*z = 20*
Y‐Z Plane Group B	[a,0,z; −a,0,z; a,0,−z; −a,0,−z]	5 (B5)	B5‐I	B5‐II	B5‐III	B5‐IV
		10 (B10)	B10‐I	B10‐II	B10‐III	B10‐IV
		15 (B15)	B15‐I	B15‐II	B15‐III	B15‐IV
Cross Group C	[a,a,z; −a,−a,z; a,a,−z; −a,−a,−z]	5 (C5)	C5‐I	C5‐II	C5‐III	C5‐IV
		10 (C10)	C10‐I	C10‐II	C10‐III	C10‐IV
		15 (C15)	C15‐I	C15‐II	C15‐III	C15‐IV
			*z = 1*	*z = 3*	*z = 5*	*z = 6.67*
Scattered Group D	[0,0,3z;a,a,z;−a,−a,−z;0,0,−3z]	5 (D5)	D5‐I	D5‐II	D5‐III	D5‐IV
		10 (D10)	D10‐I	D10‐II	D10‐III	D10‐IV
		15 (D15)	D15‐I	D15‐II	D15‐III	D15‐IV

### 2.B Prostate plans

Total of 101 previously treated prostate VMAT cases, containing 202 arcs were used in this study. All plans were created using Varian Eclipse treatment planning system (Varian Medical Systems, Inc., Palo Alto, CA, USA). The plans were chosen such that the target was limited to the prostate with corresponding PTV margin only.

### 2.C Overlapping ratio

The projected fiducial marker locations that overlap at a particular EPID projection will reduce the detectability of the prostate at that EPID projection angle, therefore, the overlap ratio was introduced to take into account the reduction of prostate detectability due to overlap of projected fiducial marker locations on the EPID images. The overlapping ratio was defined as the ratio of number of control points having overlap of projected fiducial markers on EPID images to the total control points in an arc.

### 2.D Detectability score calculation

A particular fiducial marker is considered to be detectable if the area comprising a 3 mm radius around the corresponding fiducial marker center was unblocked by the MLC or the jaws. The unblocked area $\left(A_{u}^{\mathrm{ij}}\right)$ is calculated for each fiducial marker *i* at control point *j*. The detectability score was calculated as the percentage of the summed unblocked area over the total area (*A*) for all fiducial markers at control points *j*.

The detectability of the prostate location on an EPID image is greatly affected by the number of fiducial markers detected on that image. A detection‐number weighting factor (*w*_*d*_) was introduced to penalize the control points that had less than three unblocked fiducial markers detected (a valid fiducial marker detection has an area larger than 3/4 of its original area unblocked, which is 3/16 of A). The *w*_*d*_ weighting factor was set to 1 if three or more markers were visible on a projected EPID image for a particular control point. The factor is set to 2/3 for only two marker detections, 1/3 for only one marker detection, and zero if no markers were detected.

In addition, an overlap weighting factor (*w*_*o*_) was incorporated into the detectability score to take into account the overlapped markers on the projected EPID images. This factor is different from the aforementioned overlapping ratio in two aspects: (a) it was applied only for the unblocked markers while the overlapping ratio was calculated for the open field, and (b) for each control point, the total number of overlapped markers are counted and treated differently. The *w*_*o*_ weighting factor was set to 1 if there is no overlap between all four fiducial markers, *w*_*o*_ was set to 0.5 when two markers overlap, and 1/3 for all other cases.

The fiducial markers that were blocked by the MLC or jaws for consecutive control points were additionally penalized in the detectability score with a weighting factor (*w*_*ts*_). *w*_*ts*_ was given for the entire arc. Each segment of consecutive control points that had no detection was first counted. The threshold of 15 control points was given to sift out the segments that were not deemed as large time interval. One minus the half of summed control points of all remaining segments over the total number of control points was the final *w*_*ts*_ value.

Thus, the detectability score (*D*) was calculated as:

(1) $$D=w_{\mathrm{ts}}\sum_{j}^{n}w_{d}w_{o}\frac{\sum_{i}^{4}A_{u}^{\mathrm{ij}}}{A}$$

where *i* is the fiducial marker number, *j* is control point number, *n* corresponds to the total number of control points for that arc, *w*_*d*_ is the weighting factor of detectable number of markers, *w*_*o*_ is the overlapping weighting factor, *w*_*ts*_ is the time span weighing factor, $A_{u}^{\mathrm{ij}}$ is the unblocked area for *i* ^*th*^ fiducial marker at control point *j*, and *A* is the total area of the four fiducial markers.

### 2.E Optimal distance analysis

The detectability score was determined for the 40 fiducial marker implant patterns illustrated in Table 1 and Fig. 1. An optimal distance from the center of the prostate to implant the fiducial markers was determined based on the detectability score results. The markers were assumed to be distributed as a circular pattern in X‐Y plane. The shortest radius to the center was assumed to be the separation distance of the X‐Y plane. Along the Z direction, the score was evaluated via shortest separation distance between marks; if the markers happened to share the same Z value, they were considered as not separated at all.

### 2.F The Modulation Complexity Score

The Modulation Complexity Score (MCS) value was calculated for each arc of all the prostate plans. The calculation method was based on the equation described by McNiven.^14^ Analysis was performed between the MCS and the detectability to see the correlation between complexity of the MLC motion and the fiducial marker detection.

## 3 RESULTS

**Figure 2:**
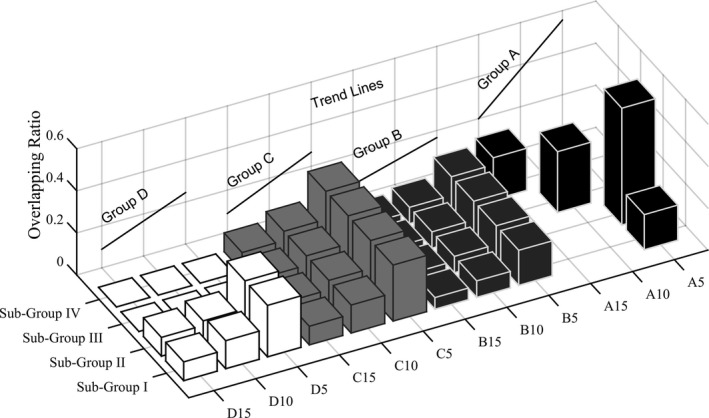
Results of Overlapping ratio, a general trend line of each group was projected onto the side plane.

As shown in Fig. 2, within each group (group A, B, C, and D), the overlapping ratio decreases as fiducial markers become more separated in the X‐Y plane (indicated by the 5, 10, 15 suffix), indicating that markers are distinguished from each other more as their separation increases in the X‐Y plane. The overlapping ratio remains constant when the fiducial markers are scattered further away from each other along the Z direction (indicated by the subgroup, from I to IV, gradually moving away from the central axis along the Z direction) until there is no overlap (group D, subgroup III and IV).

**Figure 3:**
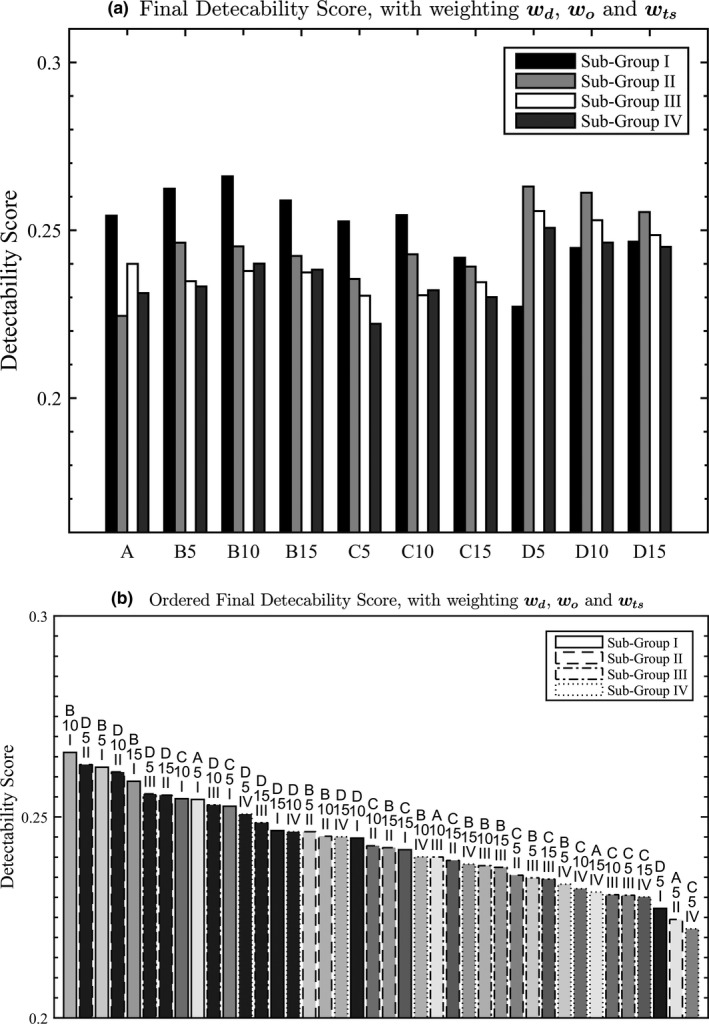
(a) Final detectability score with all the weighting factors applied and (b) Color‐coded results of the final detectability score in decreasing order. Scattered group D was highlighted in the same dark gray, while all the other groups were colored in different scale of gray.

The overall detectability scores are shown in Fig. 3. In general, the detectability score decreases as the fiducial markers get more separated along the superior–inferior (Z) direction (comparing subgroup I to IV, the average detectability score decreased from 0.268 to 0.221). Subgroups II, III, and IV in the scattered group D show high detectability scores compared to the other groups. The group D is characterized by having marker locations that are more scattered from each other than the other groups. The groups A and C, on average, had the lowest detectability score. If the results were sorted in order, as shown in Fig. 3(b), the marker positions in group D occupied the most area in the high detectability region.

**Figure 4:**
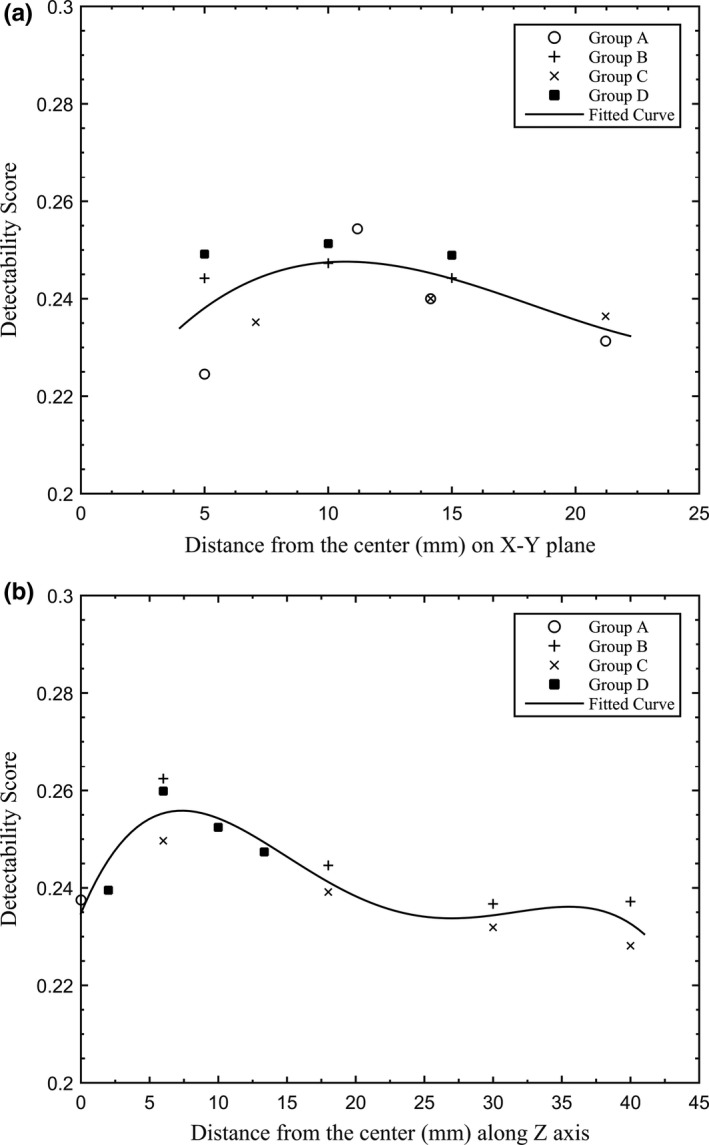
Optimal distance results: (a) X‐Y plane separation; (b) Z direction interval.

Figure 4 shows the detectability score as a function of fiducial marker location (distance) from the central axis (CAX). Figure 4(a) displays the score as a function of distance in the X‐Y plane while Fig. 4(b) shows the score as a function of distance in the Z direction. The detectability score remains constant from the 10 mm fiducial marker location to 15 mm in the X‐Y plane. Beyond 20 mm, the detectability of the fiducial markers decreases, indicating that as the markers are placed beyond 20 mm from the CAX in the X‐Y plane, the detectability of the prostate decreases. The optimum detectability occurs when the markers are placed between 6 and 13 mm from CAX in the superior–inferior direction. If additional markers are positioned at the peripheral region, they should be place twice as far away as the markers close to the CAX. The reason is groups A, B, and C have only two Z values, which means every two fiducial markers share one transverse plane. But group D has four different Z coordinates for each marker, hence they will be detected more than the markers in groups A, B, and C.

**Figure 5:**
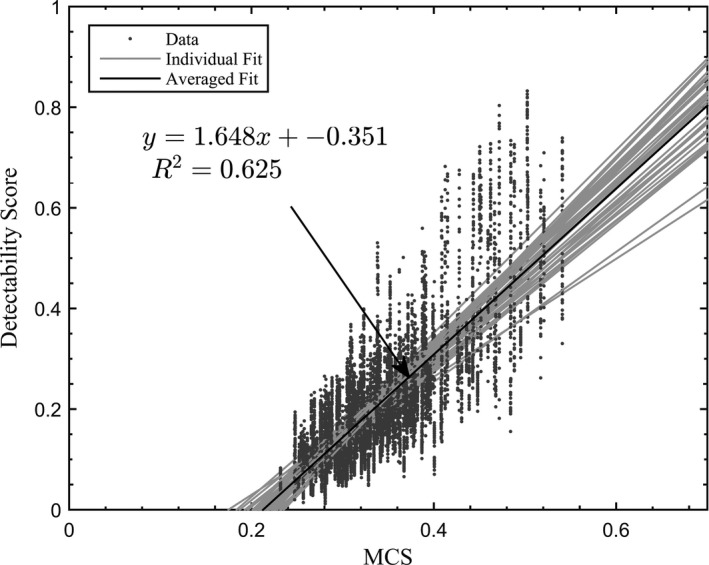
Relationship between detectability score and MCS.

Figure 5 shows the linear relationship between MCS and the detectability score. The highly modulated plans (low MCS scores) result in a low detectability score, indicating that complexity of the plan also is a factor in the detectability of the prostate during treatments.

## 4 DISCUSSION

As seen from Fig. 2, the overlapping ratio decreases as markers are separated in the X‐Y plane. The ratio will drop to 0 in the superior–inferior (Z) direction if the separation interval between two marker centers is greater than the length of one fiducial marker; otherwise it will remain constant. These results indicate that the markers should be placed as far away from each other in each direction to avoid overlap in the projected EPID images.

Figures 3 and 4 show that the fiducial markers are best detected when placed between 10 and 15 mm from the center of the prostate (assuming the CAX is placed at the center of the prostate) in the X‐Y plane and between 6 and 13 mm from the center of the prostate in the superior–inferior direction. Fiducial markers placed beyond 20 mm will have low detectability on the projected EPID images. This can be explained by the fact that in a typical arc field, the dynamic MLC is more likely to be open around the CAX. The markers placed around the center are less likely to be blocked by the MLC, hence will be detected more in the projected EPID images. The markers placed toward the periphery of the PTV (beyond 20 mm from the center of the prostate) will have reduced detectability since they are mostly blocked by the MLC.

Meanwhile, placing the fiducial markers closer to the CAX means that the changes limited to the very peripheral region of the prostate would not be detected, especially the rotational changes of the prostate. It is a balance between detecting most of the markers in the projected EPID images to determine the position of the prostate during the entire treatment and accurately determining the rotation of the prostate during treatment. The optimum placement of the markers should be toward the higher limit of the proposed range to maximize the detectability of the markers and to accurately monitor rotational changes in the prostate.

**Figure 6:**
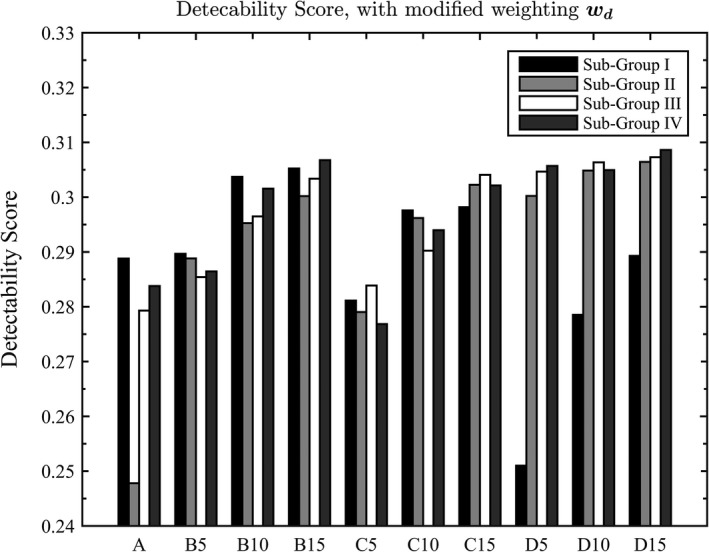
Final detectability score for new detection‐number weighting factor.

In certain clinical environment, one marker is considered sufficient enough to pinpoint the target location at a time. Then, the detection‐number weighting factor would be assigned to 1 for cases that at least one valid detection is observed and 0 for no detection. To take these situations into account, a recalculation of the detectability score with the new detection weighting factor was presented here in Fig. 6.

The detectability score would tend to favor the ones scattered more on the X‐Y plane. This is reasonable. Majorly, the MLC openings are around the central region. The MLC will irradiate occasionally around the peripheral area, which, with no detection number penalty, gives a boost to detectability score in the original scores. Compared to the original results, the more peripheral the markers get, the higher the chance will be blocked. Therefore, with the original detect number weighting factor, the patterns with more scattered markers got lower values. Whether to choose the original format and simple detection‐number weighting format is purely depended on how it is going to be implemented in the clinical environment. However, the final results remain the same.

The relationship between MCS and detectability indicated that lower complexity of the treatment field would improve the overall performance of the marker tracking technique via MV imager. Future work can be done to maintain the same treatment plan quality while decreasing the complexity of the MLC movements.

**Figure 7:**
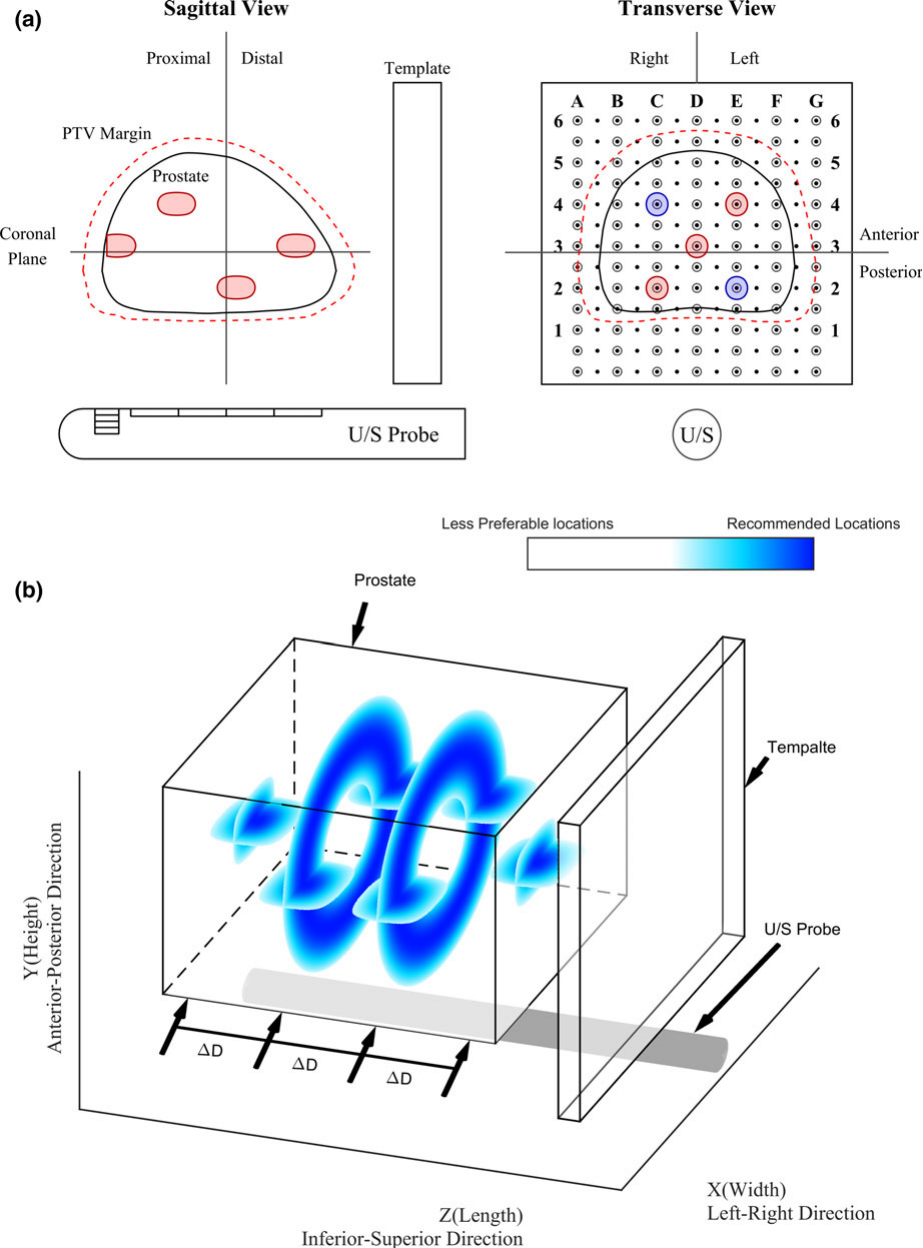
(a) Example of suggested marker locations, red color was the planted position, while blue ones could be an alternative for the two marker locations in the middle. The prostate is contour in solid black, while the PTV is contoured in dotted red line (b) Highlight of the suggested locations from the proposed method, where Δ*D* is the interval between each plane.

Figures 7(a) and 7(b) show the recommended marker deposition locations in 2D and 3D, respectively. The relative location of grid template and the ultrasound probe were labeled on each graph. The distribution depicted in Fig. 7(a) is a combination of transverse view on the template plane and a sagittal view on the central prostate plane for visualization in ultrasound guidance. The four markers in red on the sagittal view are the suggested pattern for the inserted fiducial markers. Due to the nature of symmetry, the two markers closer to the central line can also be implanted at locations in blue on the transverse view. In addition to the 2D demonstration, a 3D distribution is shown in Fig. 7(b) via five different planes (four transverse planes and one central coronal plane) to directly illustrate the possible spatial distribution and orientation of these fiducial markers in a real prostate. More importantly, the figure gives a clear indication about the spatial interval between each marker along the Z direction. Δ*D* designates the interval between each transverse plane 6–13 mm is recommended for Δ*D*. The color from dark blue to white (transparent) indicated the degree of preference from highly recommended to not‐recommended marker placement. Dark blue indicates the recommended fiducial marker location bands/spots. The rate gradually decreased as the position moves away from the central dark blue region.

## 5 CONCLUSIONS

Tracking the movement of the prostate is crucial during treatment to ensure the intended dose is given to the target while minimizing the dose to surrounding healthy organs. From our results, the optimal locations to place the fiducial markers are determined to be 10–15 mm from the center of the prostate in the transverse plane and 6–13 mm in the superior–inferior direction. These fiducial marker locations will enable the best detectability of the prostate to provide better real‐time tracking with the MV imaging technique.

## CONFLICT OF INTEREST

The authors have no other relevant conflicts of interest to disclose.
